# Inclusion health patient perspectives on remote access to general practice: a qualitative study

**DOI:** 10.3399/BJGPO.2023.0023

**Published:** 2023-04-19

**Authors:** Aaminah Verity, Victoria Tzortziou Brown

**Affiliations:** 1 Wolfson Institute of Population Health, Queen Mary University of London, London, UK

**Keywords:** general practice, technology, health policy

## Abstract

**Background:**

The COVID-19 pandemic has led to rapid and widespread adoption of remote consultations and triage-first pathways in general practice. However, there is a lack of evidence on how these changes have been perceived by patients from inclusion health groups.

**Aim:**

To explore the perspectives of individuals from inclusion health groups on the provision and accessibility of remote general practice services.

**Design & setting:**

A qualitative study with individuals from Gypsy, Roma and Traveller communities, sex workers, vulnerable migrants, and those experiencing homelessness, recruited by Healthwatch in east London.

**Method:**

The study materials were co-produced with people with lived experience of social exclusion. Semi-structured interviews with 21 participants were audiorecorded, transcribed, and analysed using the framework method.

**Results:**

Analysis identified barriers to access owing to lack of translation availability, digital exclusion, and a complex healthcare system, which is difficult to navigate. The role of triage and general practice in emergencies often seemed unclear to participants. Other themes identified included the importance of trust, face-to-face consultation options for ensuring safety, and the benefits of remote access, particularly in terms of convenience and saving time. Themes on reducing barriers included improving staff capacity and communication, offering tailored options and continuity of care, and simplifying care processes.

**Conclusion:**

The study highlighted the importance of a tailored approach for addressing the multiple barriers to care for inclusion health groups and the need for clearer and inclusive communication on the available triage and care pathways.

## How this fits in

The COVID-19 pandemic resulted in a rapid uptake of digital and remote ways of working within general practice. Digital technologies have often been presented as central to the delivery of a fairer healthcare system, and a digital NHS is being promoted by policymakers. However, there are also concerns about introducing a ‘digital divide’, which can exacerbate existing health inequalities and access barriers to care for already excluded and vulnerable groups of patients. This qualitative study explored how the shift to remote general practice care affected access for people from a wide range of inclusion health groups. The findings show the additional barriers that a remote-by-default approach may result in and highlights the importance of flexible, responsive, and tailored care pathways, and of clear, compassionate, and inclusive communication.

## Introduction

Inclusion health is a term used to describe people who are socially excluded, are affected by multiple risk factors for poor health, and experience stigma and discrimination.^1^ Evidence shows that people from inclusion health groups face barriers in accessing and engaging with health and care services, underuse primary and preventive care, and often rely on emergency services when their health needs become acute.^2–5^ Health services are not accommodating to the language, cultural, and practical needs of this population,^6,7^ exacerbating the unfair and avoidable differences in health, and resulting in poor health outcomes and significantly higher than average mortality rates.^8^

A rights-based approach to health requires that health policy and programmes must prioritise the needs of those furthest behind towards greater equity.^9^ The need to address health inequalities has received growing attention within health research and policy settings.^10,11^ Concurrently, digital health technologies have grown and been positioned by policymakers as central to the delivery of a fairer healthcare system.^12^ The pandemic catalysed the widespread adoption of remote ways of working within the NHS, including general practice. ‘Total triage’, where all patient requests for GP consultations required some form of triage, and ‘remote-by-default’ consulting, where a clinician should consult remotely (by online, phone, or video consultation) unless there is a 'clinical exception', were mandated^13^ to reduce COVID-19 transmission. There are calls for greater expansion and implementation of such remote ways of working;^14,15^ however, digital technologies have been identified as a new determinant of health and there are concerns that they can exacerbate existing inequalities unless carefully governed and implemented.^16,17^

There is limited evidence on the barriers to GP access and the impact of the recent changes on inclusion health populations, with existing evidence mainly focusing on migrants and people experiencing homelessness.^18,19^ This study aimed to explore the perspectives of people from a wider range of inclusion health groups, bringing to light not only the access issues these patients face but also their suggestions for improvements. The findings can guide work on improving the implementation of digital and remote ways of working within general practice and the experience of care for these populations.

## Method

Participatory qualitative research methodology was employed in two urban case study sites (Newham and Tower Hamlets) from June 2021 until January 2022.

### Co-production

The study protocol and materials were co-produced with people with lived experience of social exclusion (experts by experience [EbEs]), including experience of homelessness, asylum seeking, sex work, drug addiction, and being a member of the Romani community, to ensure relevance, sensitivity, and applicability.^20^ Pathway (Faculty for Homeless and Inclusion Health) facilitated this co-production work, the outputs of which included the participant information sheet, consent form, interview questions, advert for recruitment, and study protocol. Interview questions focused on experience of accessing general practice before and during the pandemic to try and understand the impact of triaging and remote consulting on inclusion health populations. Topics of interest were as follows: a) access to GP care; b) experience of remote interactions; and c) suggestions for improved care.

### Qualitative data collection

Healthwatch Tower Hamlets and Healthwatch Newham were recruited through the Healthwatch national team to work with the study research team and perform semi-structured interviews with local service users. Healthwatch's statutory role is finding out what communities want from health and social care, and it has a research governance framework on how to collect qualitative data and obtain informed consent in vulnerable groups. The consent processes, data storage and handling, and participant incentives were agreed in line with the study's ethical approval. Regular meetings were held with the data collection teams to ensure adherence to the study protocol and troubleshoot problems around data collection.

There were two data collection teams, one under Healthwatch Tower Hamlets consisting of three researchers and one from Healthwatch Newham consisting of two researchers. Healthwatch branches used their local expertise to identify local support organisations for inclusion health groups to facilitate the recruitment of study participants. Communications and engagement materials were in lay language and translation services were used when needed. Individual and group interviews were performed in a mixture of face-to-face and remote settings depending on the accessibility needs and preferences of participants. Participants were not known to the interviewers. Interviewees were reimbursed £30 for their time. Interviews were audiorecorded and transcribed, and the anonymised transcripts were shared with the research team for data analysis.

### Analysis

The framework method was used for the analysis to allow important themes to be identified with the co-production group, which consisted of non-experts in data analysis.^21^ Researchers who had collected the data from Healthwatch were asked to build a coding framework given their familiarisation with the data from performing and transcribing the interviews. The two coding frameworks, one from Healthwatch Tower Hamlets and one from Healthwatch Newham, were then amalgamated into a single framework. The co-production group met and reviewed the framework against a sample of three transcripts and undertook a critical analysis, which resulted in further iteration of the main themes. This process led to a final co-produced framework, which guided the qualitative analysis of the transcripts during the indexing and charting phases.

## Results

### Sample characteristics

In total, 21 participants were interviewed (Table 1). Tower Hamlets undertook eight interviews with 11 participants and Newham 10 interviews with 10 participants. All of Newham’s interviews were one-to-one interviews but three of them included support workers providing translation. Six of Tower Hamlets’ interviews were one-to-one interviews. One was with two participants and one was with three participants who requested to be interviewed as groups. Three of the Tower Hamlet interviews used translators who were present with the participants at the time of interview. The recruitment period was 4 months and challenges were faced recruiting interviewees who were sex workers and from Gypsy, Roma, and Traveller communities.

**Table 1.** Characteristics of interview participants, *N* = 21

Characteristic		% (*n*)
**Background**	Experiencing homelessness	24 (5)
	Sex worker	24 (5)
	Vulnerable migrant	38 (8)
	Gypsy, Roma, and Traveller community	14 (3)
**Age, years**	18–30	29 (6)
	31–50	48 (10)
	51–70	24 (5)
**Sex**	Male	38 (8)
	Female	62 (13)
	Prefer not to say	0 (0)
	Other	0 (0)

### Experiences of GP access

The key themes identified with illustrative quotes are presented in Table 2.

**Table 2.** Experiences of GP access: themes and illustrative quotes

Experiences of GP access
**Reflections on waiting times and GP role in emergencies**
*'I had to wait one week, if I was lucky, one week.'* (Person experiencing homelessness)*'You need to wait; you have to wait. It’s OK, it’s fine. I think it’s fine.'* (Refugee)*'Actually, all GPs are different, so the first GP I was not liked. Because you call now even if you are emergency, even if you said like I have to be seen by a doctor, they book appointment after one week. The second GP, it was nice. They booked appointment in one day.'* (Refugee)*'So, the GP doesn’t tackle any emergencies. Even though when I call them and I says I have the jaw problem, he says why did you not go to the emergency?'* (Person experiencing homelessness)*'When she had COVID I called the GP because she had a problem with breathing, and they called me in one hour with a video consultation.'* (Vulnerable migrant)
**Complex booking process**
*'Then you try to make it more complicated by telling me to go call this number and this call number doesn’t link to the answer you need, the communication and speaking with my GP and services are blocked off.'* (Person experiencing homelessness)
**Feelings around GP access**
*'And my doctor doesn’t judge you. Because I needs to be comfortable with somebody. If I feels like someone’s judging me, I won’t go back to see them.'* (Sex worker)*'But when you feel like you’re constantly ringing them and can’t get through it feels like you’re always on the phone to them. And it’s very … frustrating, really frustrating it is.'* (Vulnerable migrant)*'Didn’t bother making an appointment as it`s difficult to make an appointment.'* (Person experiencing homelessness)*'If anything happened to the boy, they would blame me and tell me I should’ve taken him to the GP, which I tried to do but they didn’t respond.'* (Vulnerable migrant)
**Understanding of GP challenges**
*'Well, I would give the GP a job, but I wouldn’t give the system a job. So, you don’t blame the GP, you blame the system.'* (Traveller)*'It doesn’t matter for waiting, because I know he’s working hard, too many people is calling.'* (Refugee)

Most responders expressed a positive experience with GP access pre-pandemic compared with post-pandemic. Post-pandemic, the main negative experiences quoted were long waits on the phone for appointments and difficulties accessing face-to-face appointments.

Some responders, however, felt positive about access to GP care post-pandemic, commenting on fast response times and easy access.

There was divergence of opinion on what were appropriate waiting times, with some participants thinking that 1 week's waiting was excessive. There was also some confusion on whether GP services should deal with emergencies. One-third of responders described the post-pandemic appointment booking process as complex.

Several participants reported feeling respected and not judged, but others described feeling anxious and frustrated when trying to access GP care in the post-pandemic period. A few participants' experiences of GP access had led to them feeling apathetic about accessing primary care while others expressed fear of being blamed if they did not seek GP advice.

Several participants expressed an understanding of the challenges GP practices face.

### Barriers to access

The themes that emerged are presented in Table 3.

**Table 3.** Barriers to GP access: themes and illustrative quotes

Barriers to access
**Digital skills**
*'They just said read the website and gave me a pin number, but the pin number doesn’t work. Website A, has a password that doesn’t work for website B, more jargon, then C doesn’t work, E doesn’t work.'* (Person experiencing homelessness)*'Yeah, some people, I’ve got a friend at home now, he doesn’t know how to do anything on the internet. He asks me, can you book me an appointment, do this, do that, and I’ll help him out. But he doesn’t know how to do it, does he?'* (Person experiencing homelessness)
**Digital access**
*'So the doctor, the GP, is sending you text messages, but you’re unable to answer them because you don’t have money on your phone to do it.'* (Vulnerable migrant)*'My phone is a £10 phone, and they keep saying show me a photograph. I say listen, I haven’t got one of them phones.'* (Traveller)*'It says on the website to book online but he doesn’t have an account, he doesn’t have access to the internet.'* (Person experiencing homelessness with support worker)*'Because if I download, if I send them images, yeah my internet is not enough internet for me.' (*Traveller*)*
**Language and/or cultural barriers**
*'It was really difficult because it’s press one, press two, press this, press this, I didn’t understand nothing ... And after this I need to ask for interpreter, for example, but they make many questions. When I came in the beginning, I didn’t know this, how to spell. And I didn’t understand how important it is here to spell names and surnames and things like this.'* (Vulnerable migrant)*'And I need to specify I need an interpreter and a female doctor, female interpreter.'* (Vulnerable migrant)
**Registration barriers**
*'I asked if I can be registered, and she said no you have to use the internet to make an appointment, and this can’t be changed*.*'* (Person experiencing homelessness)*'Because to register, you need to have an ID, and I was waiting for the first interview by the Home Office, and I didn’t have ID*.*'* (Vulnerable migrant)
**Complexity of the healthcare system**
*'You couldn’t get the doctor … in hospital, when I called them 111, they told me to go A&E yeah. After A&E, then the GP out of hours … '* (Vulnerable migrant)*'Can I book an appointment doctor? No, you have to go online consultation. How do I do that? So, I’ll try and do it and it goes back. You press one button; it brings you back. It rings 999. But I didn’t have that much of a pain to ring 999.'* (Traveller)

Digital exclusion included not having the skills and/or ability to access digital technology. Low income was described by several participants as a barrier to engaging with remote consulting, specifically maintaining a working phone. Accessing remote and online consulting were considered more challenging with a language barrier. Nine participants said they had no difficulties with accessing free Wi-Fi and getting online. However, most participants mentioned needing an advocate or peer support to use digital tools to access GP care, either because of language or digital literacy issues. Problems with translators were reported, including lack of availability, and needing to rely on friends and/or family to access GP care.

Participants also mentioned difficulties with registration, including not having proof of identification or address, moving often, and digital-only registration options. Several responders commented on the complexity of the healthcare system with unclear pathways of care.

### Triage system

Only six responders were able to describe the triage process at their practice. One participant described a digital-first triage model, and five responders described a receptionist-based triage system.

More than half of the responders required interviewers to explain the term 'triage' to them and how it would apply in practice. Three responders felt that triage was not necessary in the context of general practice because people should not be presenting for emergency care, and therefore everyone should wait the same amount of time. In addition, some responders felt it was inappropriate to have to share their situation with the receptionist, preferring to speak directly to the GP. However, several of the responders felt that triage was necessary and helped GPs prioritise and sort appointment requests. There was an understanding expressed that not all queries needed face-to-face appointments and of the importance of introducing triage-first models during the pandemic to reduce COVID-19 transmission.

Table 4 summarises the themes generated.

**Table 4.** GP triage: themes and illustrative quotes

Triage system
**Triage helps prioritisation**
*'Yes, depending. For example, if I have just a question about a prescription, I don’t have to go to the GP for a prescription. I can call and ask.' (*Sex worker)*'I can say they can give first priority who is vulnerable, more vulnerable.'* (Vulnerable migrant)
**Triage is not necessary**
*'I never called the GP for fun! Yeah, I called because I have issues.'* (Traveller)
**Triage requires interpreters**
*'Sometimes I will phone, and I ask can we translate as they don’t understand and I don’t understand, over the phone appointment. They don’t give a translator and I tell them I don’t understand, and this isn’t OK as I am sick.'* (Person experiencing homelessness)
**Triage by receptionists**
*'I think receptionist makes an appointment, isn’t it? So, when they see vulnerable people, when they see a circumstance, they can easily find who is vulnerable and who is not.'* (Vulnerable migrant)*'When people say to her could you tell me what’s wrong with you today? I’m here to see the doctor, I’m not here to see you.'* (Traveller)*'...* [they need to] *do their job properly eg, reception not acting as if they are a doctor.'* (Person experiencing homelessness)

### Digital access and remote consulting

Table 5 presents the positive and negative themes identified when asking about GP digital access and remote consulting. Eleven responders acknowledged positive aspects including convenience, enabling tailored and effective care, and protecting from infection. One-third of responders felt that remote consultations could lead to a trusted GP assessment, especially when there is continuity of care.

**Table 5.** Positive and negative themes on digital access and illustrative quotes

Digital access and remote consulting
**Positive themes**
**Convenience**
*'Even with the prescription one, it’s simple. I’m not that tech good, it’s just like name, date, it’s simple, and I’m not very good at them things.'* (Sex worker)*'It’s much easier, because it’s not easy to move, walking so long distance, just to be wasting so much time on it.'* (Person experiencing homelessness)*'I don’t want to sit in the waiting room waiting, and then go in there just for a prescription, when they can do it on the phone. If there’s nothing wrong with me, and I don’t need a check-over, it’s just the same thing, just the tablets.'* (Sex worker)
**Enabling tailored care**
*'But it was quick. Doctor saw the e-consultation and gave an appointment face to face.'* (Vulnerable migrant)*'Because I’ve learnt now if you have a rash you take a picture and send it to the doctor. So, the doctor knows what he’s talking about.*' (Sex worker)
**Enabling effective care, especially when there is continuity**
*'If you understand it, it’s fine. If you understand everything doctor talk to you, it’s fine, because doctor he knows your problem, he knows your everything.'* (Person experiencing homelessness)
**Protecting from infection**
*'At the time I thought it was safer because I didn’t want to go in and make my GP sick, so I had to think about it and what to do to make it safer for my GP.'* (Traveller)
**Negative themes**
**Communication barriers**
*'Because sometimes you may not be able to write what you want to write on there on the internet, you aren’t able to write better than you could explain and see face to face.'* (Traveller)*'For homeless people I think it’s much preferable to be in person than on the phone, because even though you can find a phone on the street and you can call from those phones, some of them work, some of them doesn’t work, and the problem with those ones is too much noise and you can’t really hear properly, and they can’t hear you properly.'* (Person experiencing homelessness)*'So you’re trying to rush down everything you need to say and you’ll miss certain things out and then think after the phone call I haven’t said that.'* (Sex worker)*'I didn’t understand what they were talking about. They didn’t seem to be understanding me because they weren’t looking at my face.'* (Traveller)
**Practical considerations: confidentiality**
*'Yes. If my children … any children, hear about their mother’s illness, they feel upset. I don't want a consultation in front of my children.'* (Vulnerable migrant)
**Perceptions of care inefficiency**
*'If I do e-consult yeah, doctor will take 48 hours. It’s about three days to call me back. Within three days, yeah, I should call 111 because I need to see a doctor, that’s why I call 111. Because I can’t wait three days!'* (Vulnerable migrant)*'Maybe online talk to another one, call another one, text another one. Maybe doctors talk to another one* [at the same time]*.'* (Vulnerable migrant)
**Concerns about safety**
*'It was different because when I see my GP face to face, they can test me and do regular blood check-up but because it’s over the phone they can’t really do any tests like blood pressure, stepping onto the scales to see how much I weigh, they can’t do this when I’m over the phone.'* (Asylum seeker)*'I used to go to the GP. But since the COVID she’s talking on the phone to me, what’s your problem? She can’t see my problem, I’m trying to tell her what’s wrong with me, but she can’t see it so how would she know what was wrong with me?'* (Traveller)*'It’s not good because you need to check the temperature, look at the eyes, listen to the lungs and see if something is there*.*'* (Vulnerable migrant)
**Harder to build trust**
*'Everything was just calls, calls, all calls, no connection whatsoever and a lot of issues emerged from it, I know for sure.'* (Person experiencing homelessness)*'Because online I’m talking to another person. I’m writing, he not see me. He’s not listening. Maybe he don’t listen. Half an ear.'* (Refugee)*'So all this stuff about when you phone are they there, it’s important but actually that’s not the point is it? No, it’s about being in the doctors’. It’s about being with your doctor.'* (Sex worker)

Sixteen participants expressed negative views around remote consulting. Several participants felt that they couldn’t make themselves understood during remote consultations and language barriers were exacerbated, while some also felt more pressured and rushed. Several participants were concerned that if they spoke English as a second language, they could be misdiagnosed based on a remote assessment.

Practical considerations around remote consulting included the lack of private space for holding the remote consultation. In addition, some participants mentioned worsening care efficiency as a result of digitalisation.

More than half of responders felt that remote consultations did not provide the same experience as face-to-face appointments, and several of those found it harder to build trust with clinicians remotely. Several participants felt that face-to-face appointments are needed to provide safe and effective care, with an emphasis on physical examination being key to diagnosis.

### Reducing barriers

When participants were asked about how to reduce barriers to GP access they made several suggestions, which are summarised in Table 6 with key themes.

**Table 6.** Reducing barriers to GP access: themes and illustrative quotes

Reducing barriers
**Increasing staff capacity**
**More staff at reception**
*'Pick up the phones, get extra people in to answer the phone to give someone peace of mind. Because if they don’t answer the phone, it’s like you’re blocked out.'* (Traveller)
**Using multidisciplinary team to improve system capacity**
*'Long-term disease, you’re just passing those renewal to the chemist, and then you can have like a review every six months to see if something improves it, if something changed and so on, so by doing so, I think you remove at least 20, 30% of the people that come into the GP.'* (Vulnerable migrant)
**Tailored options to care**
**Alternative non-digital routes for booking appointments**
*'Making appointments over the phone as it is easier to make them like this instead of over the internet.'* (Person experiencing homelessness)*'So, you call round, and they say yes we can make an appointment. Yeah, that would be a lot easier.'* (Traveller)*'Feels GP services should be like how they were before lockdown and be able to book an appointment and be seen straightaway.'* (Vulnerable migrant)
**Face-to-face appointment options**
*'For now, every GP you go to it is the same thing, if they can change the system, it will be very good to make it how it was before so that they can check you properly.'* (Person experiencing homelessness)
**More flexibility with appointment times**
*'Yeah, you can go half an hour before time and you’re still sitting there. You go one minute late; they make you another appointment. It’s true.'* (Traveller)
**Continuity of care**
*'I like to see the same one, because if I go in today with a spot, he knows what he’s looking for. The next time if it’s going away or if it’s getting bigger, if it’s going smaller. A new doctor wouldn’t know, would he?'* (Traveller)*'Yes, because for my health issues, always it was the same doctor, always the same female doctor. Yes, and you start having confidence when you don’t need to repeat the same things.'* (Vulnerable migrant)*'That’s why I contacted my GP because doctor knows better than us; isn’t it?'* (Person Experiencing homelessness)
**Simplifying care pathways**
*'Make it much easier to see them rather than pressing A, B, using websites. Just make it simple like phone, or even walking in, just make it easy.'* (Person experiencing homelessness)
**Improved communication**
**Interpreters at triage stage**
*'If you don’t speak English, you don’t know English, maybe it’s better just to call them and say interpreter please — only this word. But actually, the first call, they don’t give this option of interpreter, I don’t know, I’m not sure, but I don’t think they give this option of interpreter in the first call with reception.'* (Vulnerable migrant)
**Communication of service changes**
*'Would it help to have notes about what’s changed and what hasn’t changed? Yeah, that would be helpful.'* (Person experiencing homelessness)
**Compassionate and approachable staff attitude**
*'Ring someone back and tell them you’re expecting a phone call. You may expect a phone call at such a time between what hours. Might be on the hour because sometimes you miss a call, they might be busy with the next person. Just give them that bit of time.'* (Traveller)*'I’d say look I know I’m struggling and that, I’m ever so sorry and that, but can you help me just sort this out. Please can I just talk to somebody or something like that, and just try and ask for a tiny bit of support.'* (Sex worker)

Practical system changes suggested were increasing staff capacity, a tailored approach to care, simplified care processes, offering choice of remote and face-to-face options and appointment flexibility, and the availability of interpreters during triage. Continuity of care seemed to be valued by most responders.

Participants also suggested finding ways to better communicate service changes and ensure a compassionate and approachable staff attitude.

## Discussion

### Summary

This study explored the experiences of people from inclusion health groups on access to and use of GP services during the biggest shift to remote care provision. It also identified the barriers to accessing and receiving care, and the potential solutions from the perspectives of service users. The rapid move to GP total triage and remote-by-default consulting seemed to have exacerbated pre-existing barriers to access owing to language issues and difficulty navigating the already complex healthcare system. It introduced new barriers owing to digital illiteracy and challenges around privacy, maintaining a good WI-FI connection, and having a working camera phone. Participants indicated this has led to an increasing reliance on others, such as interpreters, support workers, and family members, to navigate the system. There was a perception that face-to-face appointments are needed to provide safe and effective care. Continuity of care seemed to be particularly valued by people from inclusion groups and was recommended as a way of reducing access barriers. The potential benefits of remote access were also recognised, particularly in terms of convenience and saving time, with a preference for telephone over digital ways of communicating. Participants highlighted the importance of a compassionate attitude and a need for a clearer articulation of the available healthcare access points, triage, care pathways, and the role of general practice in emergencies.

### Strengths and limitations

The study used co-production methods in its design, and local stakeholders and outreach workers to recruit a diverse group of inclusion health participants. As a result, it managed to capture a wide range of experiences from vulnerable groups of patients who are generally underrepresented in primary care research. Collaborating with Healthwatch, an expert organisation in collecting patient feedback, to undertake the qualitative interviews introduced more rigour and independence, and allowed the study to expand to possible solutions rather than just focusing on identifying barriers to care.

It is acknowledged that the study findings did not include voices from all inclusion health groups. For example, it did not specifically focus on patients experiencing drug and alcohol dependence, partly because of the additional primary care services providing care for these patients. However, it should be noted that interview participants were not homogenous groups representing a single inclusion health group, and although they were identified because of one characteristic linked to their support organisation or local network, several of them reported experiencing multiple causes of social exclusion, including drug and alcohol dependence.

### Comparison with existing literature

Several studies have assessed the acceptability, feasibility, efficacy, and practicality of remote consulting, and showed potential benefits to patients and clinicians, but also revealed a higher uptake by younger, more affluent people and raised concerns on its safety and cost-effectiveness.^22–27^

Very few studies have explored the impact specifically on inclusion health groups, and those who did found similar themes to the present study including the difficulty of patients’ building trust and rapport, difficulty maintaining the infrastructure needed for remote consulting, the need for advocates to navigate booking appointments, exacerbation of language barriers, and concerns about the safety and effectiveness of remote clinical assessments.^17,18,28,29^

Continuity and trust were mentioned as important and linked to safety by several participants and this is in accordance with other studies.^30,31^

Participants' perceptions that face-to-face appointments are needed to provide safe and effective care, with an emphasis on physical examination often being key to diagnosis, seems to be shared by clinicians in other studies.^25,32^ However, for appropriate populations, especially once trust is established, remote consultations can be safe and effective.^31^

### Implications for practice

This study highlights common barriers to care experienced by people from inclusion groups and the variation in experiences, highlighting the importance of a flexible, responsive, and tailored approach to care.

The availability of consultation options needs to coincide with a simplification of care pathways and clarification of the role of general practice for urgent and emergency situations.

Other practical suggestions from study participants included access to interpreters at triage stage and availability of advocates, support workers, and care coordinators who can assist people with difficulties navigating the system. This would require resources and improved system capacity.

The importance of trust, respect, and relational continuity for people from vulnerable groups has been highlighted in this and other studies, and is something that will need to be incorporated when designing new ways of working and models of care.

Clarity of the role of triage and reassurance on confidentiality seemed to be important to participants who expressed ambivalence about discussing their health needs with non-clinicians. Staff training on inclusion health may also help to enhance trust and remove barriers to care.

The findings have shown that digital and remote technologies can potentially advance social justice and reduce health inequalities if they are co-designed with service users and implemented with ethical principles and human rights-based approaches in mind.
